# Alpha‐synuclein seeding amplification assay use and potential impact in a behavioral neurology clinic

**DOI:** 10.1002/alz70856_106298

**Published:** 2026-01-08

**Authors:** D. Luke Fischer, Tara N. Ellingson, Bruce L. Miller, Lawren VandeVrede

**Affiliations:** ^1^ Memory and Aging Center, Weill Institute for Neurosciences, University of California, San Francisco, San Francisco, CA, USA

## Abstract

**Background:**

The cerebrospinal fluid (CSF) SAA has high specificity for Lewy body disease (LBD) in well‐defined research cohorts, but real‐world use data are sparse. In persons with Alzheimer's disease markers, SAA positivity correlates with worse cognitive decline and may alter expectations for amyloid‐targeting drugs’ effectiveness. The study objective was to characterize current use and potential impact for clinical decision‐making of the alpha‐synuclein seeding amplification assay (SAA) in a tertiary behavioral neurology clinic.

**Method:**

Two retrospective cohorts were derived from all cases who underwent clinical lumbar puncture: 1) those with a commercial SAA test (Amprion) ordered clinically, and 2) those with CSF stored for research and SAA obtained later. Records were reviewed for diagnosis, clinical syndrome/affected cognitive domain(s), cognitive tests, presence of core LBD features and other symptoms, MRI, PET, and SPECT imaging, Alzheimer's disease biofluid markers, tests ordered by the clinician, and subsequent clinical decisions.

**Result:**

In the first year, the SAA was performed clinically for 21 patients (cohort 1). Most were men (76%). Patients at time of testing were between 53‐86 years old (mean 74). Mild cognitive impairment was the most common severity (71%) followed by dementia (19%) and subjective cognitive impairment or normal (9.5%). The SAA was abnormal in 9 patients (43%). The p(181)‐tau/amyloid‐beta(42) ratio was abnormal in 44% of SAA‐positive cases and 45% of SAA‐negative cases. In SAA‐positive cases, 5 met the core features from McKeith et al. for probable and 2 for possible LBD syndrome; in SAA‐negative cases, none were probable and 7 were possible. Seventeen had questionable or mild motor parkinsonism. Clinical syndromes varied and included amnestic, multidomain (2/6 positive), non‐amnestic, dysexecutive and behavioral (0/5), logopenic primary progressive aphasia (1/1), corticobasal syndrome (1/1), behavioral variant frontotemporal dementia (0/1), multiple system atrophy (0/1), and Parkinson's disease (0/1); no purely amnestic patients were tested. Subsequent clinical decisions included additional tests and medication changes (lecanemab included). In cohort 2, SAA was positive in 12 of 50 cases (24%) with similar analyses to be completed soon.

**Conclusion:**

Implementation of SAA was successful and used in patients with or without DLB. Meeting possible DLB criteria was poorly predictive, whereas probable DLB was accurate.

